# Chloride intracellular channel protein 2: prognostic marker and correlation with PD-1/PD-L1 in breast cancer

**DOI:** 10.18632/aging.103712

**Published:** 2020-09-11

**Authors:** Tao Xu, Zhi Wang, Menglu Dong, Di Wu, Shujie Liao, Xingrui Li

**Affiliations:** 1 Department of Thyroid and Breast Surgery, Tongji Hospital, Tongji Medical College of HUST, Wuhan 430030, Hubei, People’s Republic of China; 2 Department of Obstetrics and Gynecology, Cancer Biology Research Center, Tongji Hospital, Tongji Medical College of HUST, Wuhan 430030, Hubei, People’s Republic of China

**Keywords:** CLIC2, breast cancer, prognosis, PD-1/PD-L1, immune infiltration

## Abstract

Immune checkpoint inhibition has emerged as an effective treatment for multiple solid tumors, including advanced-stage breast cancer (BC). During the past decade, the US Food and Drug Administration has approved a number of agents for immune checkpoint blockade (ICB). However, the limited data on monotherapy anti-tumor activity in BC underscores the need for robust predictive biomarker development. Here, we used weighted gene coexpression network analysis of genes differentially expressed between BC and normal tissue to identify genes coexpressed with programmed death-1 (PD-1) and its ligand (PD-L1). Tumor Immune Estimation Resource and Gene Expression Profiling Interaction Analysis were used to assess the relationship between gene expression and the abundance of tumor-infiltrating lymphocytes (TILs). We found that chloride intracellular channel protein 2 (CLIC2) was not only coexpressed with PD-1 and PD-L1, but its increased expression was associated with a favorable prognosis and enrichment of multiple TIL types, particularly CD8+ T cells. These results suggest that CLIC2 is a potentially useful biomarker for identifying BC patients who could benefit from ICB.

## INTRODUCTION

Breast cancer (BC) is the most common cancer diagnosed in women and is a leading cause of cancer death [1]. Breast-conserving surgery or mastectomy, as well as radiation therapy may be used as part of a treatment plan. Chemotherapy, endocrine therapy, or targeted therapies may also be used, depending on cancer type and molecular test results [2, 3]. However, BC often does not respond to traditional treatment [4]; therefore, new treatment regimens are urgently needed, especially for the basal-like BC subtype. Clinical trials conducted with immune checkpoint blockade (ICB) hold promise for improving BC treatment [5]. Accelerated US Food and Drug Administration (FDA) approval of atezolizumab, in combination with nab-paclitaxel for the treatment of metastatic triple-negative BC (TNBC), has stimulated enthusiasm for studying ICB agents [6, 7].

FDA has approved multiple ICB agents for melanoma and non-small cell lung carcinoma (NSCLC), and it has approved atezolizumab as a first line treatment for patients with metastatic TNBC [8]. In contrast to melanoma and NSCLC, however, ICB has not yet produced favorable benefits in BC. There are several possible reasons for this. (1) BC has not traditionally been considered immunogenic. Although not synonymous with the basal-like subtype, compared to melanoma or NSCLC, BC usually has a low number of tumor-infiltrating lymphocytes (TILs). (2) The low tumor mutational burden across BC subtypes reduces ability to generate neoantigens that can stimulate antitumor immune responses [9]. (3) Robust predictive biomarkers for an ICB response have not yet been established. Given the relatively low response rate to ICB monotherapy, clinical trials combining ICB with radiotherapy, chemotherapy, or oncogene-targeted therapies (NCT02926196, NCT03036488) are needed. In addition, studies that investigate robust biomarkers for identifying BC patients who could benefit from ICB will be critical.

Previous studies have shown that at the time of diagnosis, high-level TILs correlate with better outcomes in patients with nonmetastatic, basal-like cancer treated with standard therapeutic regimens [10, 11]. This improvement also occurred in ERBB2-enriched BC that received the anti-HER2 antibody trastuzumab [12]. Overall, in basal-like and ERBB2-enriched BCs, a “hot” immune microenvironment, containing enriched TILs, is associated with a better prognosis, compared with a “cold” immune microenvironment, containing few TILs [13, 14]. TILs provide more significant outcomes in patients who receive ICB agents that activates intrinsic T cells to eliminate tumor cells than in patients who receive standard therapeutic regimens [15]. Here, TILs are not limited to T cells because, in the tumor microenvironment, T-cell activation is controlled by the interplay of ligand-receptor interactions between T cells, dendritic cells (DCs), and tumor-associated macrophages (TAMs) [16]. TILs respond to ICB, owing to complex feedback loops and intertwined immune system regulation mechanisms [2, 17]. Therefore, it is promising that biomarkers that infer the quantity of TILs could help with identifying patients who could benefit from ICB.

Numerous trials have found that PD-L1-positive tumors had a higher overall response rate to ICB agents in patients with melanoma, NSCLC, Merkel cell carcinoma, renal cell carcinoma, and colorectal cancer than those with PD-L1 negative tumors [18–20]. In October 2015, PD-L1 was approved by the FDA as a predictive biomarker for patients with NSCLC when receive pembrolizumab. Published phase 1 studies reported that patients with metastatic BC, who incorporated anti–PD-L1 antibody atezolizumab or avelumab, had a higher objective response rate (ORR) and a longer overall survival (OS) when TILs positively expressed PD-L1 [15, 21]. Additional studies have found that significant relationships between PD-L1 expression in TNBC cancer cells and better ORR could not be established when incorporating atezolizumab [22]. Additionally, the scoring systems used for determining PD-L1 expression varied in different trials, which challenged the convenience of using PD-L1 as a selection marker.

Based on these results, we concluded that genes coexpressed with programmed cell death (PD-1) and PD-L1 in cancer cells could be effective biomarkers for patients who receive ICB [23]. We found that high expression of chloride intracellular channel protein 2 (CLIC2) in BC was correlated with increased expression of PD-1 and PD-L1, and that a high CLIC2 mRNA level has been associated with a favorable prognosis. In addition, a positive relationship exists between CLIC2 expression and TIL quantities across all BC subtypes.

## RESULTS

### Hub gene expression clusters with PD-1 or PD-L1

**Figure 1:**
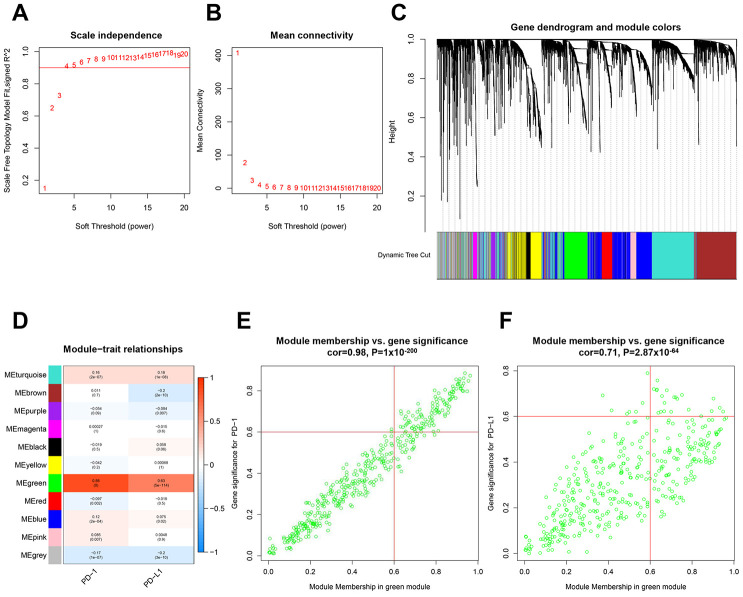
**Identification of hub gene clusters that expressed with PD-1 or PD-L1 by WGCNA.** (**A**) Plots visualize the scale-free topology fitting index. (**B**) The mean connection in different soft thresholds. (**C**) Branches of the hierarchical clustering dendrogram correspond to gene modules. (**D**) Heatmap of correlation between modules, eigengenes, and PD-1/PD-L1. Scatter plots visualize correlation between module membership in the Megreen module and gene signification for PD-1 (**E**) and PD-L1 (**F**). WGCNA, weighted gene co-expression network.

Using the limma R Bioconductor software package, a total of 3,868 differentially expressed genes (DEGs) were identified between BC and normal tissue, of which 2,688 genes upregulated and 1,180 genes downregulated in BC (Supplementary Figure 1A and 1B). Based on DEGs, a weighted gene coexpression network analysis (WGCNA) was performed to locate the gene modules in which gene expression patterns were similar. Here, we used a scale-free network distribution for the connectivity genes when the soft-threshold β value equals 4 (Figure 1A and 1B). Next, several gene modules were identified using average linkage hierarchical clustering (Figure 1C). Eleven major gene modules were identified (Figure 1D), of which the Megreen module genes positively correlated with both PD-1 (r = 0.98, p = 1 x 10^-200^) (Figure 1E) and PD-L1 (r = 0.71, p = 2.87 x 10^-64^) (Figure 1F). To reduce the scope of genes that correlated with PD-1 or PD-L1, two hub gene clusters were obtained from the Megreen module. The cluster correlated with PD-1 contained 131 genes (Supplementary Figure 2A, Supplementary Table 1), whereas the cluster correlated with PD-L1 had 37 genes (Supplementary Figure 2B, Supplementary Table 1). Using the limma packages and WGCNA, we obtained hub gene clusters that coexpressed with PD-1 or PD-L1 in BC.

### Hub gene clusters correlated with PD-1 and PD-L1 participated in immune response

**Figure 2:**
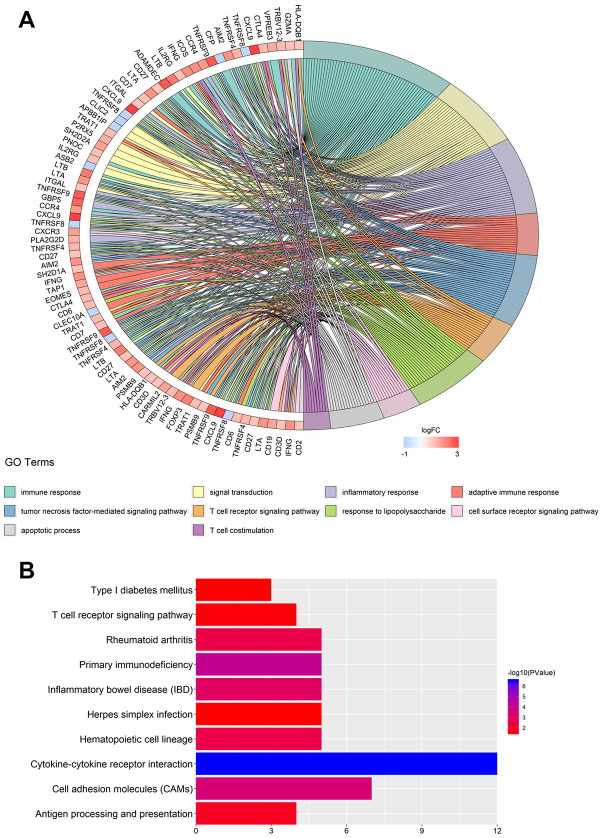
**GO term and KEGG pathway enrichment analysis for the hub gene clusters.** (**A**) Major GO terms in hub gene clusters correlated to PD-1. (**B**) Top-ranked KEGG pathways in hub gene clusters correlated to PD-1. GO, gene ontology; KEGG, Kyoto Encyclopedia of Genes and Genomes.

To further explore PD-1 and PD-L1 correlation, we mapped hub gene clusters to biologic function and enrichment analysis. The top five overrepresented Gene Ontology (GO) categories correlated to PD-1 were immune response, signal transduction, inflammatory response, adaptive immune response, and tumor necrosis factor-mediated signaling pathway (Figure 2A, Table 1). The top five enrichment pathways identified were cytokine-cytokine receptor interaction, primary immunodeficiency, cell adhesion molecules (CAMs), inflammatory bowel disease, and hematopoietic cell lineage (Figure 2B, Table 1). GO categories and enrichment analysis correlated to PD-L1, consistent with PD-1, demonstrating an association of immunity (Supplementary Figure 3A and 3B, Supplementary Table 2). The previous results demonstrate that genes correlated to PD-1 and PD-L1 primarily participate in key immune response pathways.

**Table 1:** GO and KEGG analysis of PD-1 co-expressing genes.

**Term**	**Description**	**Count**	**P-value**
GO:0006955	immune response	20	2.03E-14
GO:0007165	signal transduction	13	0.005114
GO:0006954	inflammatory response	11	6.85E-06
GO:0002250	adaptive immune response	9	3.09E-07
GO:0033209	tumor necrosis factor-mediated signaling pathway	8	9.46E-07
GO:0050852	T cell receptor signaling pathway	8	4.31E-06
GO:0032496	response to lipopolysaccharide	7	9.14E-05
GO:0007166	cell surface receptor signaling pathway	7	0.001403
GO:0006915	apoptotic process	7	0.040946
GO:0031295	T cell costimulation	6	2.54E-05
hsa04060	Cytokine-cytokine receptor interaction	12	2.99E-07
hsa05340	Primary immunodeficiency	5	6.42E-05
hsa04514	Cell adhesion molecules (CAMs)	7	0.000292
hsa05321	Inflammatory bowel disease (IBD)	5	0.000763
hsa04640	Hematopoietic cell lineage	5	0.002395
hsa05323	Rheumatoid arthritis	5	0.002498
hsa04612	Antigen processing and presentation	4	0.013188
hsa04660	T cell receptor signaling pathway	4	0.027223
hsa04940	Type I diabetes mellitus	3	0.030529
hsa05168	Herpes simplex infection	5	0.030930

### Identification of gene coexpression with PD-1 and PD-L1

**Figure 3:**
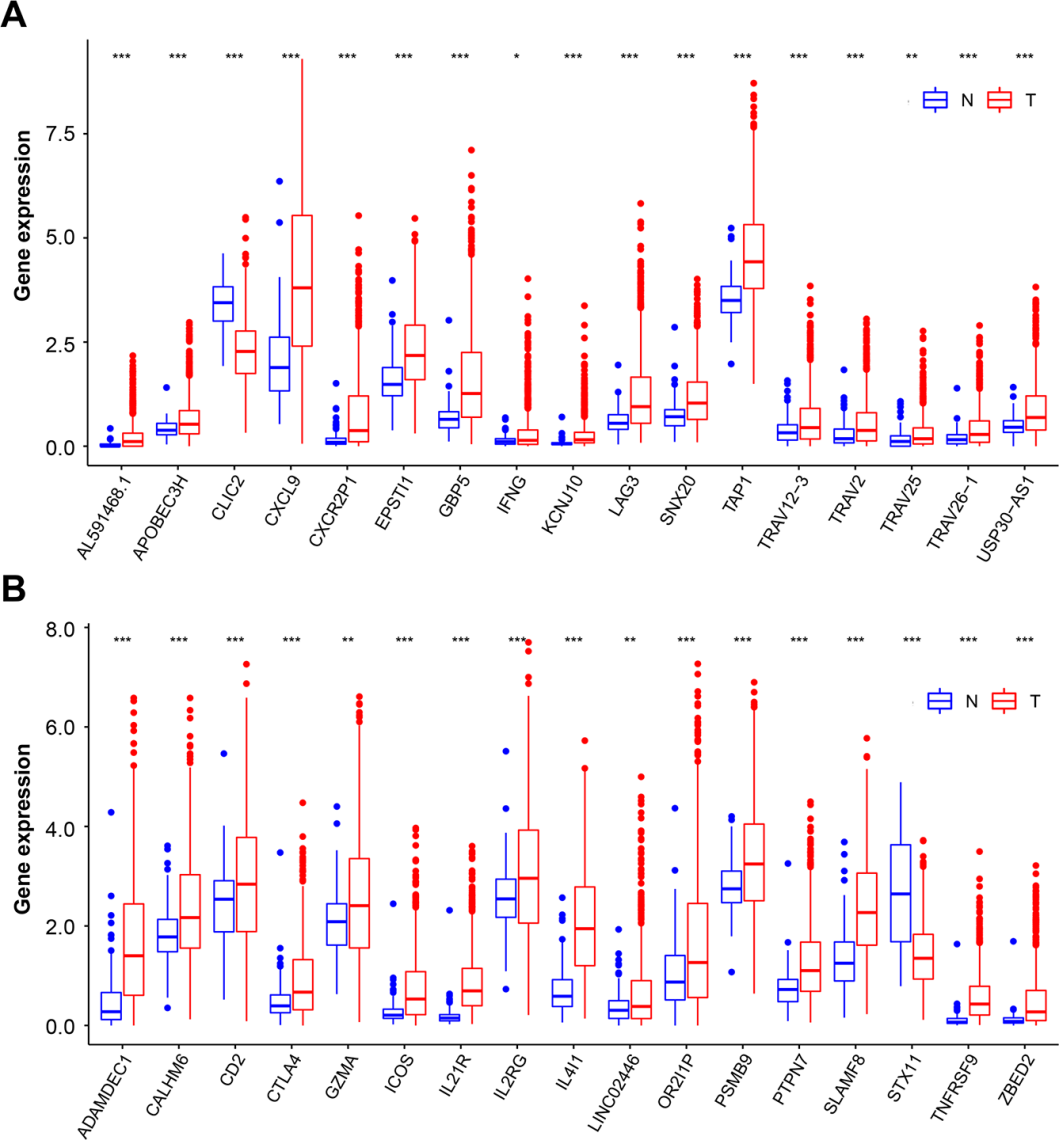
**Overlapping genes expression on BC and normal tissue samples.** (**A** and **B**) Box plot representation of 34 overlapping hub genes expression on normal tissue (blue box) and BC (red box) samples. The median, first quartile, and third quartile of expression on distribution are shown. BC, breast cancer. * p < 0.05, ** p < 0.01, *** p < 0.001.

We next tested the overlap between the two hub gene clusters, and a set of 34 genes were identified. Although most of these genes were highly expressed in BC than in normal tissue, only two genes, chloride intracellular channel protein 2 (CLIC2) and syntaxin 11 (STX11) (Supplementary Figure 2C), were lowly expressed in BC tissue (Figure 3A and 3B). CLIC2 belongs to the CLIC family of conserved metazoan proteins; however, the nature and function of CLIC2 have yet to be elucidated. It was previously reported that CLIC2 is expressed predominantly in noncancerous tissues [24]. Our results are consistent with this finding. It has previously been reported that STX11 functions as a tumor suppressor gene in peripheral T-cell lymphomas [25]. Those genes that downregulated in cancer suggest that its full function may protect cells from tumorigenesis. It is important to note that when downregulated gene expression is corrected or with agents that mimic gene function, the cancer may increase PD-L1 and PD-1 expression, leading to increased curative effect of ICB. Therefore, CLIC2 and STX11 were selected for further study.

### CLIC2 was associated with BC disease state and prognosis

**Figure 4:**
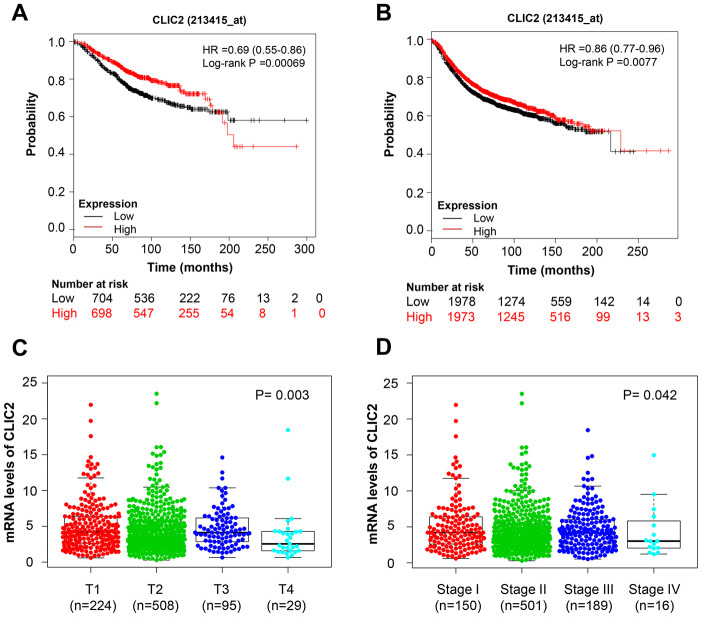
**CLIC2 was associated with BC disease state and prognosis.** Kaplan-Meier estimation of OS (**A**) and RFS (**B**) of TCGA BC patients grouped by CLIC2 expression. Gene CLIC2 expression profiles in BC with different tumor diameter (**C**) and in different stages (**D**). BC, breast cancer; OS, overall survival; PFS, progression-free survival.

To determine whether CLIC2 or STX11 was linked to BC clinical performance, Kaplan-Meier survival analysis was used on various BC subtypes. The Kaplan-Meier survival curves showed that higher CLIC2 mRNA levels were associated with longer OS (HR = 0.69 [0.55-0.86], p = 0.00069) (Figure 4A) and RFS (HR = 0.86 [0.77-0.96], p = 0.0077) (Figure 4B) in BC, whereas STX11 failed to demonstrate a significant relationship with OS (HR = 0.86 [0.69-1.06], p = 0.16) and RFS (HR = 0.92 [0.83-1.03], p = 0.14) (Supplementary Figure 4A and 4B). PrognoScan platform results showed that 5 out of 31 tests have an association between CLIC2 expression and favorable prognosis in BC, whereas other tests showed no significant correlations (Table 2). When BC cases were divided by clinical signatures (tumor size or disease stages), CLIC2 mRNA level in small tumor or early-stage cancer tissue was significantly higher than that in larger tumor or advanced-stage cancer tissue, respectively (Figure 4C and 4D). Notably, no difference in STX11 levels was observed in BC, regardless of size or stage (Supplementary Figure 4C and 4D). CLIC2 was more closely linked to clinical performance in BC than STX11. Thus, we chose CLIC2 for further study.

**Table 2:** Survival analysis of CLIC2 mRNA in breast cancer patients (the PrognoScan database).

**Dataset**	**Endpoint**	**Number**	**ln(HR-high / HR-low)**	**COX P-value**	**ln(HR)**	**HR [95% CI-low CI-up]**
GSE1456	DSS	159	-1.14	0.01	-0.42	0.66 [0.48 - 0.91]
GSE3494	DSS	236	-1.02	0.02	-0.38	0.68 [0.50 - 0.95]
GSE1456	OS	159	-1.96	0.00	-0.42	0.65 [0.50 - 0.86]
GSE7378	DSS	54	-1.50	0.04	-1.49	0.22 [0.05 - 0.93]
GSE1456	RFS	159	-0.98	0.01	-0.36	0.70 [0.53 - 0.93]

Abbreviation: DSS, Disease Specific Survival; OS, Overall Survival; RFS, Relapse Free Survival; DFS, Disease Free Survival.

### CLIC2 gene sets highly expressed cancer enriched in immune signal

**Figure 5:**
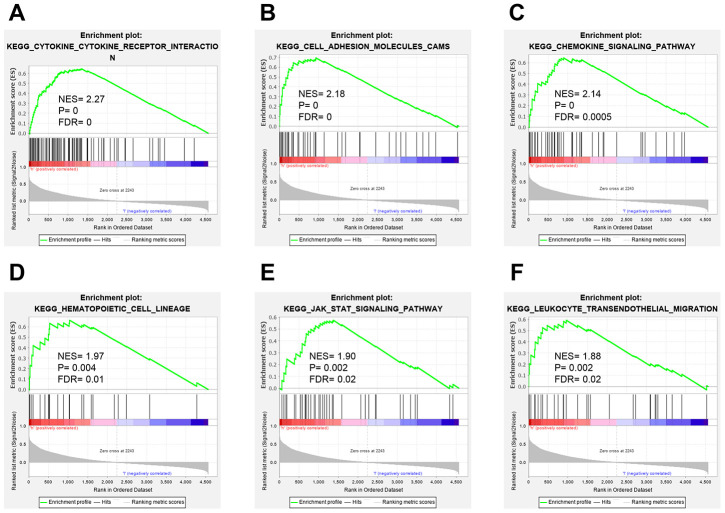
**GSEA analysis of BC grouped by CLIC2 expression.** GSEA was performed on RNA-Seq profiles of 1,053 breast cancer grouped by CLIC2 expression (high CLIC2 expression, n = 526; low CLIC2 expression, n = 527), and the top-ranked signatures were showing. (**A**) cytokine-cytokine receptor interaction; (**B**) cell adhesion molecules CAMs; (**C**) chemokine signaling pathway; (**D**) hematopoietic cell lineage; (**E**) JAK-STAT signaling pathway; (**F**) leukocyte transendothelial migration. GSEA, gene set enrichment analysis.

To our knowledge, no study has specifically investigated the role of CLIC2 on immunity function and whether immunity response is based on CLIC2 mRNA levels. Therefore, we next performed a gene set enrichment analysis (GSEA) to determine whether high expression of CLIC2 BC samples was more active in immunity. A total of 1,053 BC tissue samples were divided into two groups. We demonstrated that the CLIC2 highly expressed gene set was enriched in cytokine-cytokine receptor interaction, CAMs, chemokine signaling pathway, hematopoietic cell lineage, JAK/STAT signaling pathway, and leukocyte transendothelial migration (Figure 5A–5F). These results indicate that immunity of CLIC2 highly expressed BC is more active compared with CLIC2 lowly expressed BC.

### CLIC2 highly expressed BC was accompanied by increased TILs

**Figure 6:**
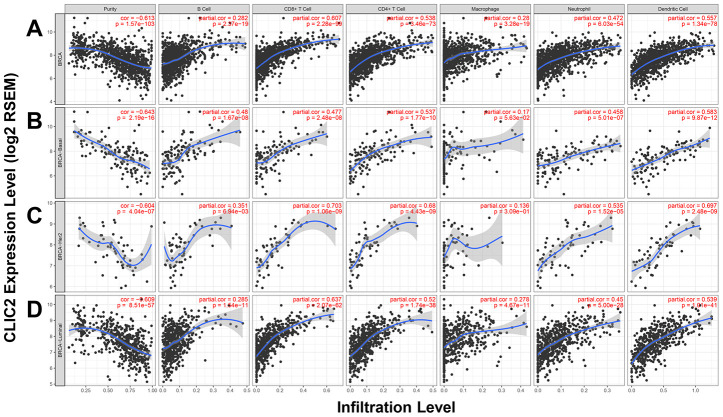
**TIMER found CLIC2 highly expressed cancer was accompanied by increased TILs.** (**A**) TIMER was performed to assess the correlation between CLIC2 expression and six types of TILs enrichment cross all subtypes of BC. Other than B cells and macrophages, CLIC2 expression has a strong positive correlation with CD8+ T cells, and moderate correlation with CD4+ T cells, neutrophils, and dendritic cells in BC. TIMER database analysis in (**B**) basal, (**C**) HER-2 and (**D**) luminal subtypes. TIMER, Tumor Immune Estimation Resource; TILs, tumor infiltration lymphocytes.

Because increased TILs improve prognosis in BC patients receiving immunotherapy, we used the Tumor Immune Estimation Resource (TIMER) web server to access the correlation between CLIC2 expression and six TIL types. Figure 6 shows that CLIC2 expression has a positive correlation with infiltrating levels of six immune cell types, including B cells, CD4+ T cells, CD8+ T cells, neutrophils, TAMs, and DCs, on total BC. Across all subtypes, CLIC2 expression positively correlated with all types of TIL enrichment in luminal cancer, whereas in basal-like cancer and HER-2 positive cancer, this relationship was also established between CLIC2 and all types of TIL enrichment, except for TAMs. This result, suggesting that CLIC2 highly expressed BC, was most likely accompanied by increasing TILs. We also examined correlation between CLIC2 expression and TILs on other tumors decreased in CLIC2 (Supplementary Figure 5). Similar results were found, particularly in lung squamous cell carcinoma (LUSC) (Supplementary Figure 6A), colon adenocarcinoma (COAD) (Supplementary Figure 6B), stomach adenocarcinoma (STAD) (Supplementary Figure 6C), and liver hepatocellular carcinoma (LIHC) (Supplementary Figure 6D). Our data showed that CLIC2 had a strong positive correlation with infiltrating levels of CD8+ T cells and DCs in these four cancer types.

### CLIC2 highly expressed cancer was accompanied by increased TIL markers

**Figure 7:**
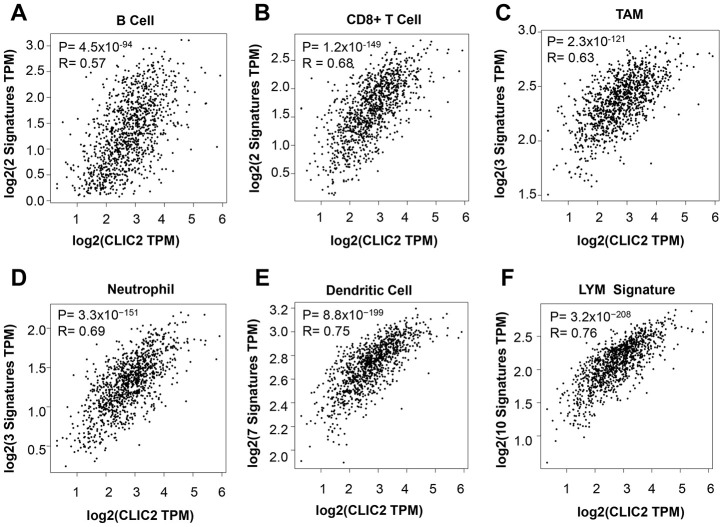
**GEPIA2 found that CLIC2 highly expressed cancer was accompanied by increased markers of TILs.** GEPIA2 was performed to assess the correlation between CLIC2 expression and markers of (**A**) CD8+ T cell, (**B**) B cell, (**C**) TAM, (**D**) neutrophil and (**E**) DC, and (**F**) LYM metagene signature. TAM, tumor- associated macrophage; DC, dendritic cell; LYM, lymphocyte-specific immune recruitment.

To confirm that increased CLIC2 mRNA levels were associated with enriched TILs, we used the Gene Expression Profiling Interaction Analysis (GEPIA2) database. Consistent with TIMER results, the GEPIA2 data showed that CLIC2 mRNA levels were positively related to the abundance of multiple immune cells, including CD8+ T cells, B cells, TAMs, neutrophils, and DCs (Figure 7A–7E). The lymphocyte-specific immune (LYM) metagene signature provided useful information for BC prognosis as well as immune phenotype [26]. We then tested whether CLIC2 mRNA levels correlate with LYM metagene signature. Figure 7F shows that CLIC2 strongly correlated with favorable LYM metagene signature. Previous GEPIA2 results support the conclusion that high CLIC2 mRNA levels in BC implied severe lymphocyte infiltration.

### Immunohistochemistry (IHC) analysis revealed correlation between CLIC2 expression and CD45+ cells

**Figure 8:**
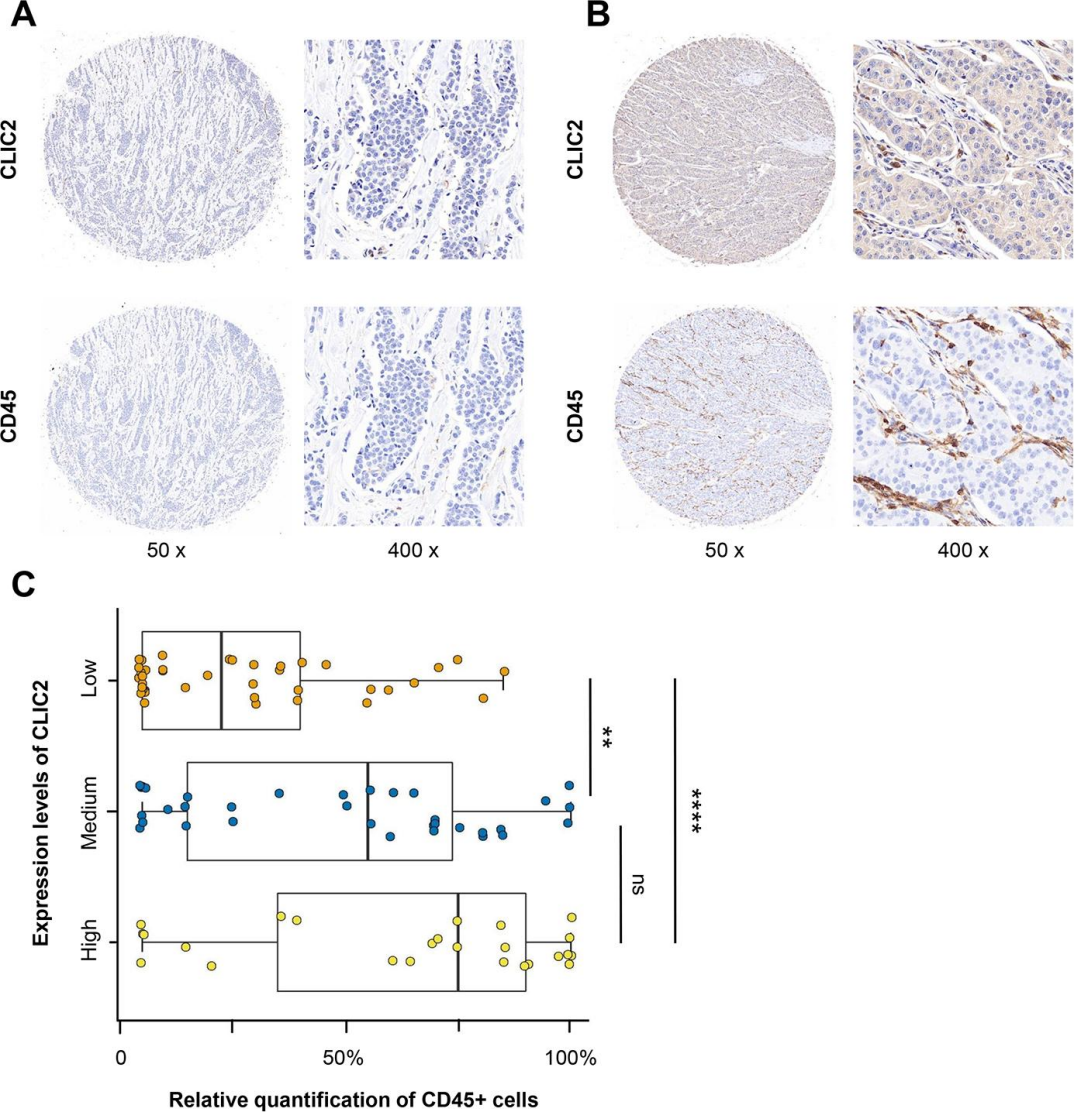
**IHC analysis found that CD45+ cells were abundant in CLIC2 highly expressed tissue.** (**A**) Low CLIC2 expression and low percentage of CD45+ cells. (**B**) High CLIC2 expression and high percentage of CD45+ cells. (**C**) Boxplot with scatter shows the different CD45+ cells percentage in tissue with different expression levels of CLIC2. ** p < 0.01, **** p < 0.0001.

We next performed large-scale tissue microarray (TMA) analysis using IHC on 99 BC tissues. Supplementary Table 3 shows the clinical characteristics of those patients. We examined and scored CLIC2 and CD45+ cell expression. Because CD45 is the common antigen that highly expressed in lymphocytes [27], we counted the anti-CD45 antibody- stained cells within stroma as TILs. Although tissue with no CLIC2 expression appeared to have rare TILs (Figure 8A), tissue with increased CLIC2 expression had infiltrating lymphocytes (Figure 8B). Significant statistical analysis showed that fewer TILs were in the CLIC2 lowly expressed group than in the CLIC2 highly expressed group (p < 0.0001) or in the CLIC2 medium expressed group (p < 0.01) (Figure 8C), thus positive correlation between CLIC2 and TIL abundance.

## DISCUSSION

Here, we identified genes differentially expressed between BC and normal tissue, which coexpressed PD-1 and PD-L1. CLIC2 and STX11 were the only two genes that substantially decreased in BC. CLIC2 encodes a chloride intracellular channel protein (CLIC), whereas STX11 encodes a member of the syntaxin family. These two protein families have distinct functions. Studies on CLIC functions in tumors are more in-depth than those focused on syntaxin function. Given that CLIC2 has higher prognostic significance on BC patients than STX11, we believe further examination of CLIC2 is needed. CLICs are highly conserved throughout metazoa, and currently six member genes have been reported [28, 29]. Among the CLICs, CLIC2 was the least investigated. Based on a previous study, CLIC2 could interact with and modulate the channel activity of RyR1 (ryanodine receptor 1) and RyR2 (ryanodine receptor 2) [30]. This indicates that CLIC2 may be a common modulator of Ca^2+^ release channels in cardiac and skeletal muscle. However, few studies have reported that CLIC2 had a physiological function in cancer or immunity. For the first time to our knowledge, our study found that CLIC2 in BC coexpressed with PD-1 and PD-L1. A recent study by Choy et al. used an unsupervised machine learning method to identify genes related to immune checkpoint proteins, including PD-1, PD-L1, and CTLA-4 [31]. According to their study, 13 genes, including CLIC2, were related to PD-1, and 26 other genes related to PD-L1. Our study provided additional evidence that CLIC2 expression is associated with PD-1. Although we found CLIC2 expression correlated with PD-L1, and Choy and colleagues failed to discover it, we attribute these findings to the different methodology that we used.

We found that high CLIC2 expression indicated a favorable prognosis in BC. However, CLIC2 mRNA expression in small tumor or early-stage cancer tissue was significantly higher than in larger tumor or advanced-stage cancer tissue, respectively. A previous study reported that CLIC2 mRNA expression at the early stage (stage IA) was significantly higher than at advanced stages (stage ≥ IB) in hepatocellular carcinoma tissues [24]. In addition, the study reported that CLIC2 predominantly expressed in endothelial cells lining blood vessels, but not lymphatic vessels, in noncancerous tissues surrounding cancer masses, in which blood vessels might be in a state of loss-of-function of CLIC2. Ueno and colleagues also indicated that CLIC2 may be involved in the maintenance of tight junctions, and that cancer vasculature endothelial lacks CLIC2, allowing the hematogenous metastasis of cancer cells. Although this provided no direct evidence that high CLIC2 mRNA level was associated with enriched TILs, cancer blood vessels with CLIC2 high expression imply relative intact function of blood vessels in the initial stage cancer. Early-stage cancer is usually more intact on immune systems than advanced-stage cancer [32, 33]. However, we have not yet ascertained whether CLIC2 high expression caused TIL abundance or if it was a signal of enriched TILs. We hope this unanswered question motivates further studies. In addition to CLIC2, several other genes coexpressed with PD-1 and PD-L1 were found to be associated with the prognosis of BC patients. For example, Kaplan-Meier analysis data showed that GBP5, EPSTI1, IFNG, KCNJ10, LAG3, and SNX20 have positive correlation with RFS in BC patients, whereas TAP has reverse correlation with RFS (data not shown).

Our results demonstrate the association between CLIC2 mRNA level and TILs. The use of GSEA showed that expressed genes in BC, with increasing CLIC2 mRNA level, enriched an important immune-related pathway. The previous results support the hypothesis that CLIC2 plays an unknown role in the immune system. In addition to BC, CLIC2 significantly downexpressed in 13 other cancer types, including bladder urothelial carcinoma, cholangiocarcinoma, COAD, kidney carcinoma, including kidney chromophobe and kidney renal papillary cell carcinoma, LIHC, lung cancer, including lung adenocarcinoma and LUSC, prostate adenocarcinoma, rectum adenocarcinoma, STAD, thyroid carcinoma, and uterine corpus endometrial carcinoma. The positive correlation between CLIC2 and abundant TILs were found on all of the previously stated carcinoma types, particularly LUSC, COAD, STAD, and LIHC. Those results indicated a common positive relationship between CLIC2 and TILs. Previously, it was reported that in LUSC, higher CD8+ TIL density was associated with longer disease-free survival [34]. Notably, pembrolizumab accompanying platinum-based chemotherapy has been approved as first-line treatment for patients with LUSC [35]. This indicated that, besides BC, higher CLIC2 expression may predict more abundant TILs and a more favorable prognosis in LUSC.

It is widely accepted that the presence of large quantities of TILs is a favorable prognostic marker in various solid tumors, including BC [11]. Clinical trial results have shown that TIL enrichment is a predictive response to the PD-1 antibody pembrolizumab, both in patients with HER2-positive BC (NCT02129556) or TNBC (KEYNOTE-086). Multifactorial markers incorporating PD-L1 expression, mutational load, intratumoral CD8+ T cells, and other variables may enhance predictive power contrast to individual tests for ICB agents [16]. Therefore, because CLIC2 coexpressed with PD-1 and PD-L1, and correlated with TIL quantities, we suggest that CLIC2 could be considered a member of predictive multifactorial markers to identify patients more likely to respond to PD-1 or PD-L1 inhibitors.

This study shows that CLIC2 coexpressed with PD-1 and PD-L1 in BC, and CLIC2 high expression was associated with a favorable prognosis and high quantities of TILs. These results suggest CLIC2 could be considered a potential biomarker to select BC patients who could derive benefit from ICB.

## MATERIALS AND METHODS

### Data collection

RNA-Seq filings along with paired clinical data of BC patients were retrieved from the publicly available TCGA (https://www.cancer.gov/tcga) database. In total, 1,053 tumor samples and 111 normal samples were collected. The RNA-seq filings were produced by Illumina HiSeq platform, and read counts were used to represent the gene expression level.

### Identification of DEGs

The R version 2.6.2 [36] was used to process gene expression data. The limma package provided by Bioconductor was used to screen the DEGs between tumor and normal samples [37]. Genes were considered as DEGs when (corrected p < 0.05 and |log2-fold-change| > 1).

### Gene coexpression network construction and module detection

WGCNA was used to construct the coexpression network and detect modules as previously described [38, 39]. Pearson correlation matrices were generated for all DEGs. Using a power adjacency function, weighted adjacency matrix of connection strengths was transformed from coexpression similarities. Next, the adjacency matrix was transformed into a topological overlap matrix to survey relative gene interconnectedness and proximity. Here, the soft threshold β value equaled 4 when the coexpression network satisfied scale-free topology. Finally, gene modules were identified based on clustering according to the topology overlap.

### Gene function and enrichment analysis

Hub genes were identified in the Megreen module when the Pearson’s correlation coefficient > 0.6 and for PD-1 or PD-L1 when the Pearson’s correlation coefficient > 0.6. GO terms and the Kyoto Encyclopedia of Genes and Genomes (KEGG) pathway enrichment analysis were performed with the hub genes cluster by means of DAVID Bioinformatics Resources 6.8 (https://david.ncifcrf.gov/) [40]. A p value < 0.05 was considered statistically significant. To demonstrate common biological pathways shared by gene set in samples with CLIC2 high expression, 1,053 BC samples were divided into two groups based on whether CLIC2 expression was greater than the median value. GSEA software was used to examine the different pathways between these two groups, as previously described [41]. Enriched terms in the KEGG pathway, with normal p < 0.05 and false discovery rate value < 0.25, were identified.

### Kaplan-Meier and PrognoSan survival analysis

The Kaplan-Meier plotter (http://kmplot.com/) [42] was used to analyze OS and progression-free survival on BC patients based on CLIC2 and STX11 expression. The patients were split by median value of gene expression. The PrognoSan database, which is publicly accessible, was examined to assess the relationship between gene expression and prognosis [43].

### Correlated analysis with CLIC2 expression to TILs

TIMER (https://cistrome.shinyapps.io/timer/) database analysis was used to examine the correlation between CLCI2 expression and TILs [44]. GEPIA2 (http://gepia.cancer-pku.cn/index.html">http://gepia.cancer-pku.cn/index.html) database analysis was used to examine the correlation between CLCI2 expression and TIL markers [45]. The gene markers of TILs referred to a previous study [45, 46], including B cells, CD8+ T cells, TAMs, neutrophils, and DCs. The LYM metagene signature referred to previous study [26].

### Immunohistochemical analysis and scoring

To examine the correlation between TILs and CLIC2 expression, IHC was performed on BC TMAs purchased from Avila (Cat No. DC-Bre11048) (Shanxi China) as previously described [47]. The anti-CD45 antibody (1:200; Catalog No. ab40763) and anti-CLIC2 antibody (1:200; Catalog No. ab175230) were purchased from Abcam. Semiquantitative scoring based on the staining intensity was used to evaluate CLIC2 expression (low, medium, and high) within the tumor cells. Anti-CD45 antibody-stained cells within the stroma were counted in the same location in which anti-CLIC2 antibody stained cells were quantified in another TMA. Scores were randomly examined by a second investigator, and an excellent intra-observer agreement was identified (k = 0.9).

### Statistical analysis

The correlation of gene expression was accessed by Spearman’s correlation coefficient. The correlations between clinical characteristics and CLIC2 expression were analyzed by a nonparametric test (the Wilcox test between two groups, and the Kruskal-Wallis test for three or more groups) [48]. In Kaplan-Meier survival analysis, p-value was calculated by multivariate Cox regression. The difference of TILs percentage of tissues on tissue microarrays was determined by using analysis of variance. A p-value less than 0.05 was considered significant.

## Supplementary Material

Supplementary Figures

Supplementary Tables
