# Supplementary material for: Chloride intracellular channel protein 2: prognostic marker and correlation with PD-1/PD-L1 in breast cancer
Source: Aging (Albany NY). 2020 Sep 11;12(17):17305–27. doi: 10.18632/aging.103712 (PMC7521498; doi:10.18632/aging.103712)
Supplement: Supplementary Figures [file aging-12-103712-s002..pdf]

SUPPLEMENTARY FIGURES

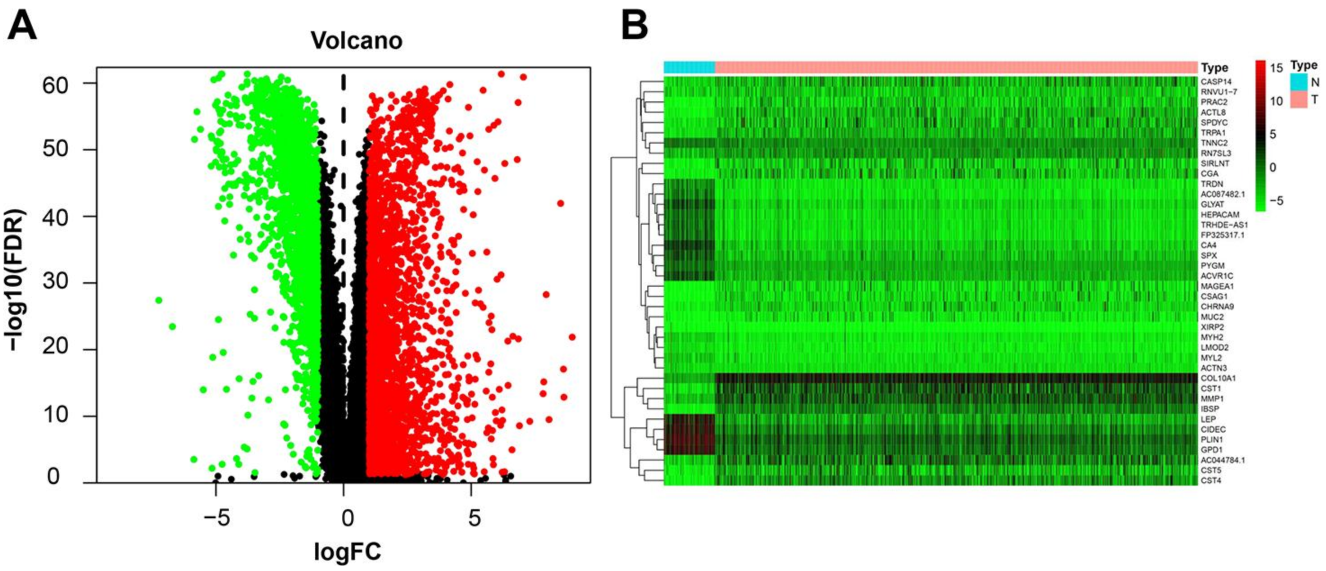

Supplementary Figure 1. DEGs between BC and normal tissue samples. (**A**) Volcano plot showing DEGs between BC (n = 1,053) and normal tissue (n = 111). Each bold dot denotes an individual gene with corrected $p < 0.05$ and |log2-fold-change (FC)| > 1. (**B**) Hierarchical clustering heatmap of DEGs. DEGs, differential expressed genes; FC, fold change.

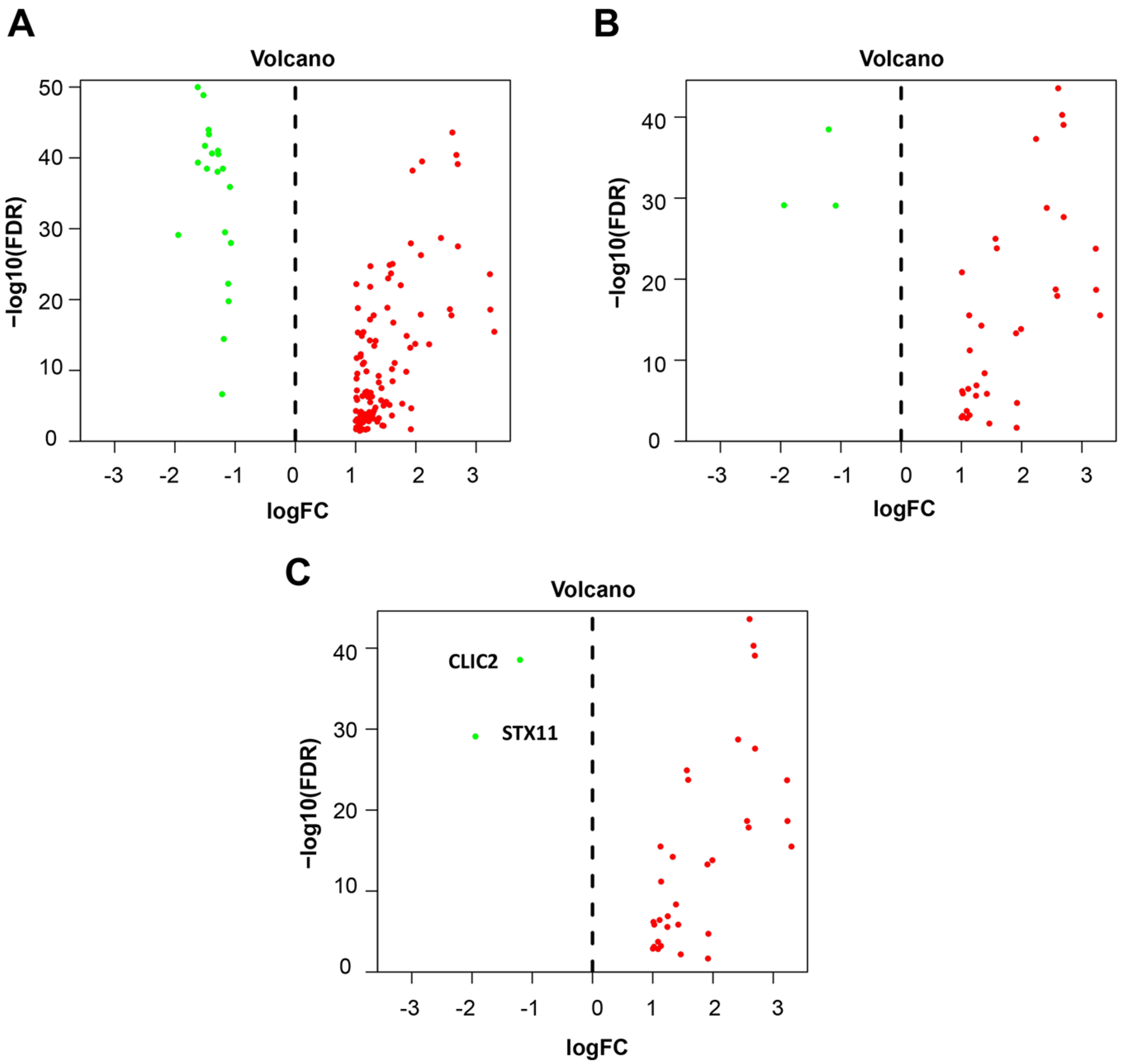

Supplementary Figure 2. Volcano plots of gene coexpression with PD-1 and PD-L1. (**A**) Volcano plot showing hub genes coexpression with PD-1. (**B**) Volcano plot showing hub genes coexpression with PD-L1. (**C**) Volcano plot showing hub genes coexpression with both PD-1 and PD-L1.

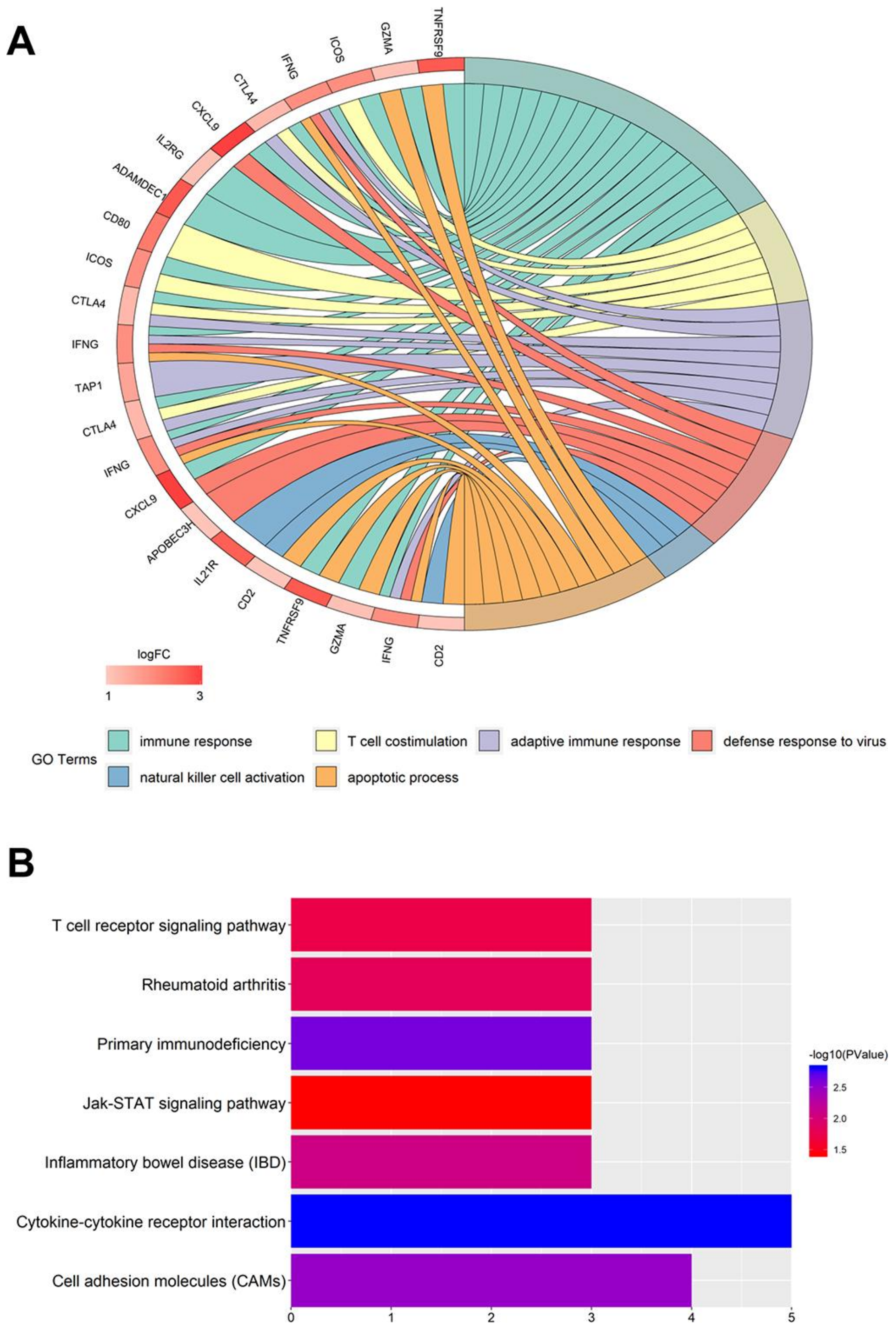

Supplementary Figure 3. GO term and KEGG pathway enrichment analysis for the hub gene cluster. (**A**) Major GO terms in hub gene clusters correlated to PD-L1 ($p < 0.05$). (**B**) Top-ranked KEGG pathways in hub gene clusters correlated to PD-L1 ($p < 0.05$). GO, gene ontology; KEGG, Kyoto Encyclopedia of Genes and Genomes.

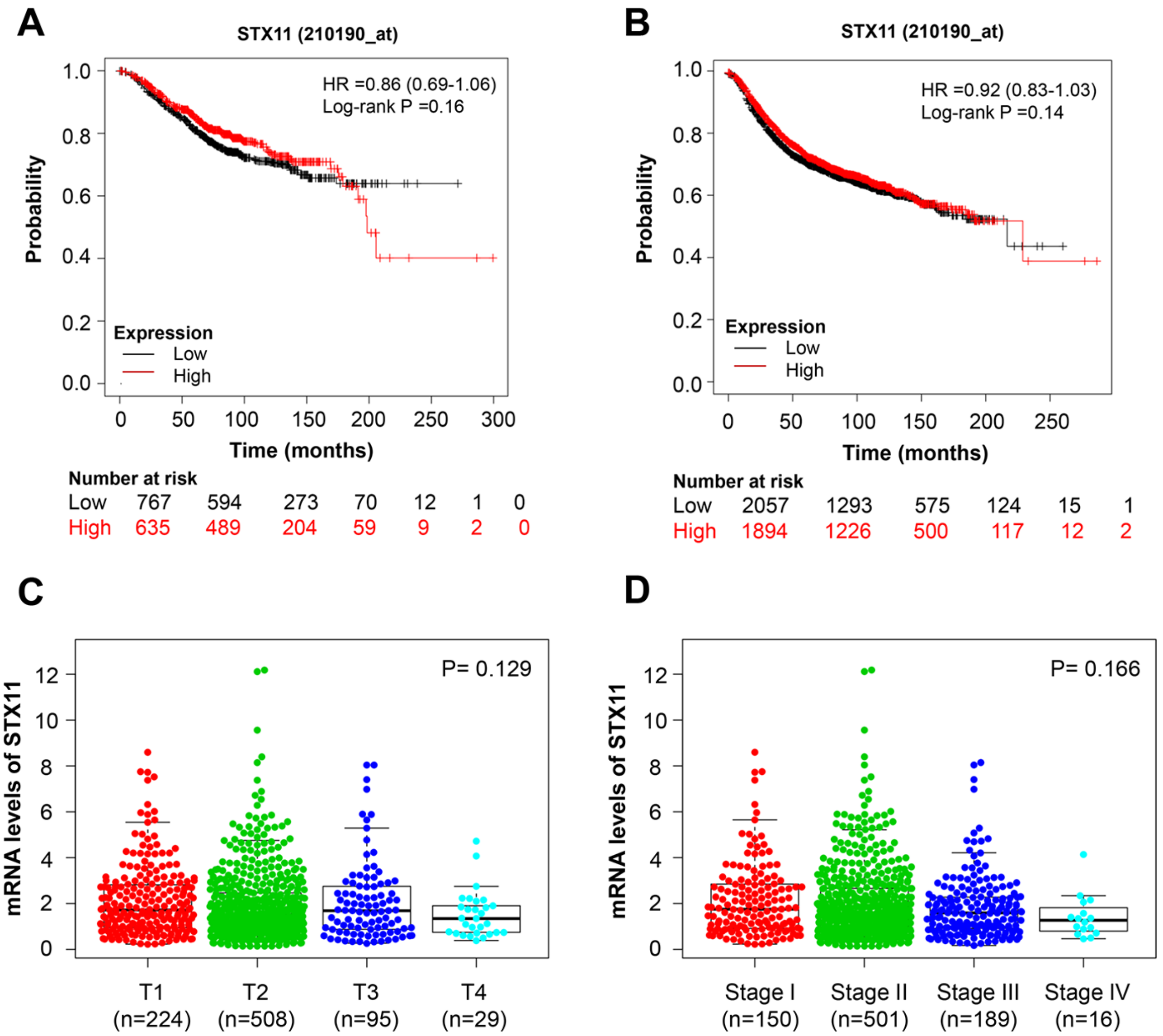

Supplementary Figure 4. The relationship between STX11 and BC clinical performance. Kaplan-Meier estimation of OS (**A**) and RFS (**B**) of TCGA BC patients grouped by STX11 expression level. Expression levels of STX11 in BC with different tumor diameter (**C**) and in different stages (**D**). BC, breast cancer; OS, overall survival; PFS, progression-free survival.

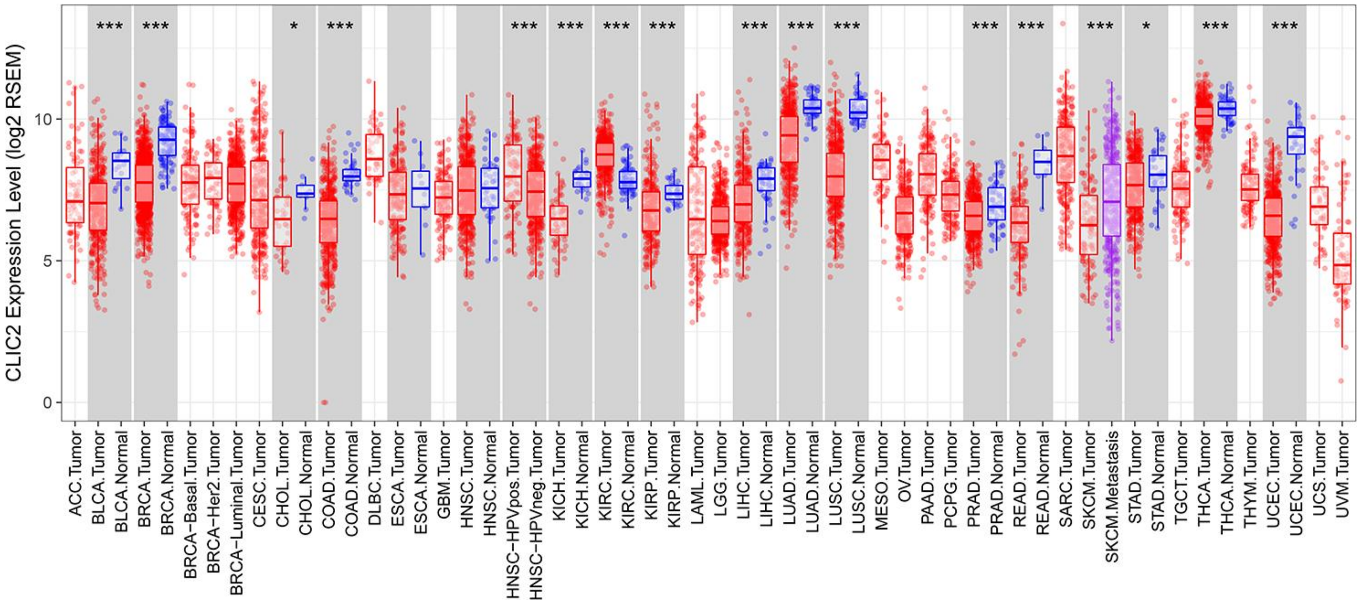

Supplementary Figure 5. CLIC2 expression levels in different cancers. CLIC2 expression in different human cancers from TCGA database were shown by TIMER database. * $p < 0.05$, *** $p < 0.001$. TCGA, The Cancer Genome Atlas; TIMER, Tumor Immune Estimation Resource.

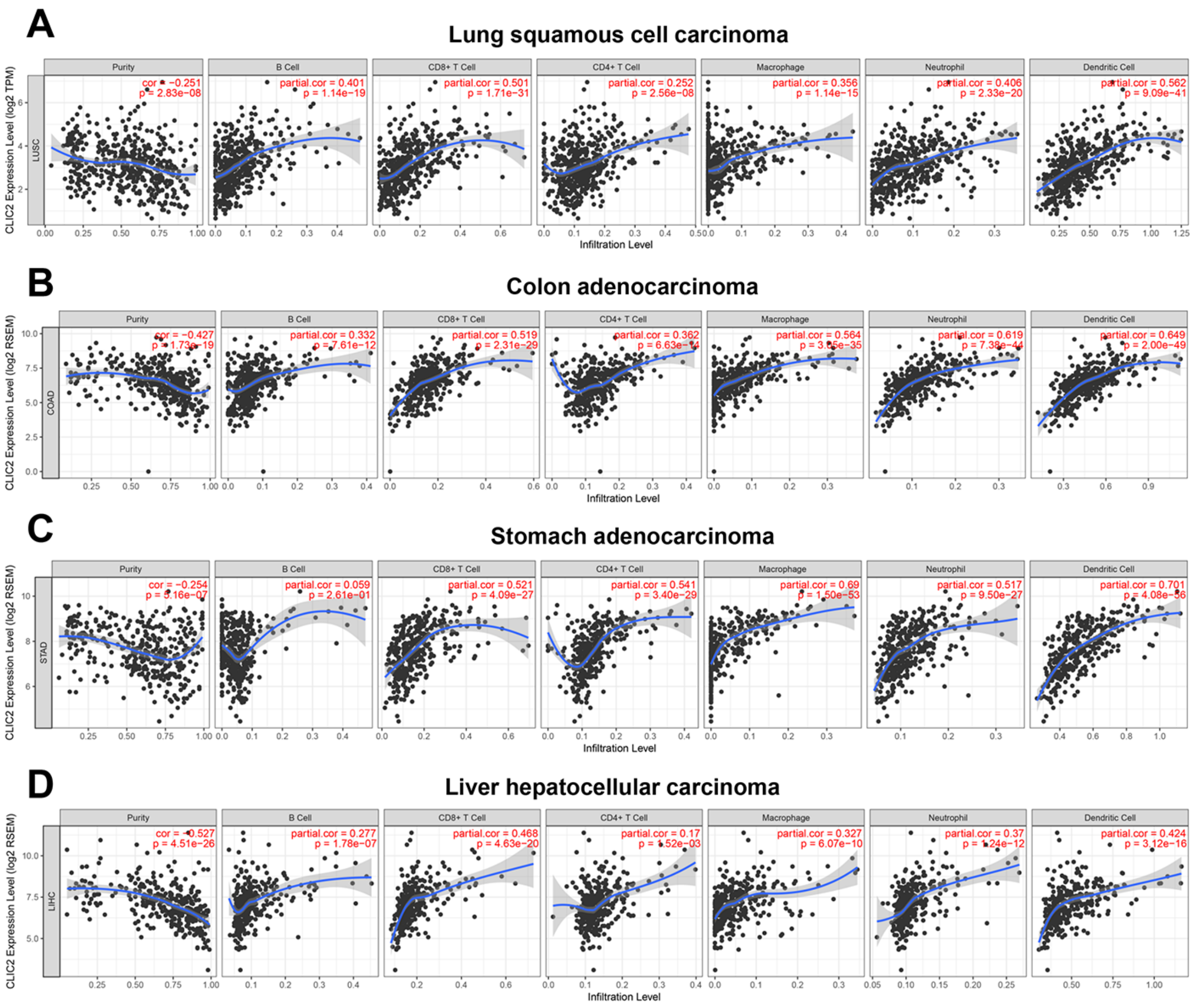

Supplementary Figure 6. CLIC2 highly expressed cancer types were accompanied by increased TILs. The TIMER database was used to assess the correlation between CLIC2 expression and six types of TILs enrichment cross four common cancers. TIMER analysis in (**A**) lung squamous cell carcinoma, (**B**) colon adenocarcinoma, (**C**) stomach adenocarcinoma, and (**D**) liver hepatocellular carcinoma. TILs, tumor infiltration lymphocytes; TIMER, Tumor Immune Estimation Resource.
