# Supplementary material for: Chloride intracellular channel protein 2: prognostic marker and correlation with PD-1/PD-L1 in breast cancer
Source: Aging (Albany NY). 2020 Sep 11;12(17):17305–27. doi: 10.18632/aging.103712 (PMC7521498; doi:10.18632/aging.103712)
Supplement: Supplementary Tables [file aging-12-103712-s001..pdf]

SUPPLEMENTARY TABLES

Supplementary Table 1. Screening PD-1/PD-L1 co-expressing genes by WGCNA method.

Description	Gene symbols
PD-L1 co-expressing genes	CLIC2 GBP5 IFNG AL591468.1 LAG3 ADAMDEC1 CXCR2P1 SLAMF8 KCNJ10 CALHM6 USP30-AS1 IL21R EPSTI1 LINC02446 TAP1 IL2RG CXCL9 ICOS CD2 IL4I1 SNX20 STX11 PSMB9 CD80 GZMA OR2I1P PTPN7 CTLA4 ZBED2 TRAV12-3 CD300LF TNFRSF9 FGL2 TRAV25 TRAV26-1 TRAV2 APOBEC3H
PD-1 co-expressing genes	CLIC2 SIRPG PTPN7 IL2RG CXCR3 CORO1A CD2 CD3D CD27 TIGIT MAP4K1 IL21R CD6 MYO1G LTA SH2D1A TRBC2 CTLA4 TRBV20-1 GZMA CD7 SP140 CD5 SNX20 EOMES TRBV28 ITGAL AC004585.1 FUT7 GZMK TRAV12-3 TRBJ2-7 TRBV6-1 ICOS TRBV5-6 TRBV18 ZBED2 TRAV41 ZC3H12D TRBV12-4 TRBV11-2 FCRL3 CXCL9 TRBV6-6 CALHM6 FOXP3 RAB33A TRAV2 APOBEC3H TRAV26-1 TRBV4-2 GIMAP7 USP30-AS1 MIR155HG TRBV15 PLA2G2D TRAV39 LAG3 TRAV25 LTB LINC02446 GIMAP1 TRBV12-3 TRAV9-2 TRBV5-5 PNOC IFNG CD19 LINC01857 IGFLR1 HLA-DQB1 APBB1IP KCNJ10 TRAV26-2 ASB2 AC004687.1 STAP1 PSMB9 LIMD2 FCRLA LINC02273 SH2D2A TNFRSF9 SHISAL2A P2RX5 IL4I1 LINC01943 GIMAP5 LINC01215 CARMIL2 SLAMF8 TRAV6 PRDM8 SOWAHD IKZF3 MIAT CXCR2P1 RHOF MCOLN2 FCMR TRBV7-3 TRAT1 CASP17P PTGDS VSIR OR2I1P CFP SLC9A9 TCL1A CCR4 STX11 GBP5 EPSTI1 FAM129C PPP1R16B FLI1 ADAMDEC1 VPREB3 GIMAP6 AL109914.1 TNFRSF4 TRDV1 AC108134.3 IFI30 RRN3P1 CLEC10A TNFRSF8 TAP1 AL591468.1 AIM2 GPR18

Supplementary Table 2. GO and KEGG analysis of PD-L1 co-expressing genes.

Term	Description	Count	P-value
GO:0006955	immune response	8	1.41757E-06
GO:0031295	T cell costimulation	3	0.005502265
GO:0002250	adaptive immune response	3	0.018757020
GO:0051607	defense response to virus	3	0.022989399
GO:0030101	natural killer cell activation	2	0.028215974
GO:0006915	apoptotic process	4	0.045793269
hsa04060	Cytokine-cytokine receptor interaction	5	0.001524288
hsa05340	Primary immunodeficiency	3	0.002391570
hsa04514	Cell adhesion molecules (CAMs)	4	0.003266245
hsa05321	Inflammatory bowel disease (IBD)	3	0.008275989
hsa05323	Rheumatoid arthritis	3	0.015247830
hsa04660	T cell receptor signaling pathway	3	0.019422333
hsa04630	JAK-STAT signaling pathway	3	0.038718075

Supplementary Table 3. Characteristics of patient subsets included in IHC analysis.

Parameters	Categories	No. of cases	Percentage (%)
Age, years (n=99)			
	20-30	1	1.01
	31-40	17	17.17
	41-50	45	45.45
	51-60	16	16.16
	61-95	20	20.20
Tumor Grade (n=98)			
	1	6	6.12
	2	71	72.45
	3	21	21.43
Tumor size, cm (n=83)			
	0.1-2.0	6	7.23
	2.1-5.0	68	81.93
	>5.0	9	10.84
Lymph node metastasis (n=99)			
	Present	24	24.24
	Absent	75	75.76
Distant Metastasis (n=99)			
	Present	0	0.00
	Absent	99	100.00
TNM Stages (n=99)			
	I	5	5.05
	II	72	72.73
	III	22	22.22
	IV	0	0.00
Molecular Subtype (n=98)			
	Luminal A	23	23.23
	Luminal B	44	44.44
	Her2 Positive	16	16.16
	TNBC	16	16.16
Relative quantification of TILs (n=99)			
	<20%	35	35.35
	20%-80%	43	43.43
	>80%	21	21.21
